# A Reciprocal Very-Low-Frequency Mechanically Resonant Magnetoelectric Antenna

**DOI:** 10.3390/ma19122652

**Published:** 2026-06-19

**Authors:** Tingyu Deng, Jinlou Gu, Dong Wang, Jie Jiao

**Affiliations:** 1 Key Laboratory for Ultrafine Materials of Ministry of Education, School of Materials Science and Engineering, East China University of Science and Technology, Shanghai 200237, China; 2 Shanghai Institute of Ceramics, Chinese Academy of Sciences, Shanghai 201800, China; wangd@mail.sic.ac.cn; 3 Center of Materials Science and Optoelectronics Engineering, University of Chinese Academy of Sciences, Beijing 100049, China; 4 University of Chinese Academy of Sciences, Beijing 100049, China

**Keywords:** mechanical magnetoelectric antenna, direct magnetoelectric effect, converse magnetoelectric effect, low-frequency wireless communication

## Abstract

This study investigates an IPS-type Metglas/PMN-PT laminated magnetoelectric composite and its feasibility as a reciprocal mechanical magnetoelectric antenna for low-frequency transmission and reception. Finite-element simulations under quasi-static and frequency-domain conditions reveal strong magnetoelectric coupling under an optimal DC bias field, with both the direct magnetoelectric effect (DME) and converse magnetoelectric effect (CME) exhibiting pronounced resonance near 14.5 kHz, governed by the same longitudinal extensional vibration mode. Five IPS samples were fabricated and experimentally characterized. All devices showed resonant frequencies within 14.1–14.5 kHz, peak DME coefficients of 3.0 × 10^6^ to 3.9 × 10^6^ pC/Oe, and peak CME coefficients of 12.0~15.8 Oe·cm/V, confirming good fabrication consistency, transmit–receive reciprocity, and array-integration potential. The parallel IPS antenna generated a magnetic flux density of 37 nT at 1 m, and exhibited an equivalent magnetic noise of 63 fT/Hz^1/2^ at 14.45 kHz. These results demonstrate that the proposed IPS structure combines high-sensitivity reception with efficient low-frequency transmission, showing strong potential for miniaturized, low-power, and long-range magnetic communication and underwater communication applications.

## 1. Introduction

Low-frequency electromagnetic communication is important for underwater communication, underground exploration, cross-media transmission, near-field wireless links, and IoT systems in special environments. However, the radiation efficiency of conventional electric antennas is strongly tied to their physical size relative to wavelength. At very-low-frequency (VLF) and ultra-low-frequency (ULF) bands, the wavelength can reach tens of kilometers or more, so efficient radiation typically requires antenna dimensions approaching one-quarter wavelength. This leads to bulky systems, high power consumption, and poor integrability [1,2,3,4,5]. Achieving efficient low-frequency transmission and highly sensitive reception within a compact form factor therefore remains a central challenge.

Antenna miniaturization in the VLF/ULF regime remains a fundamental challenge for conventional electrically small antennas, which has motivated increasing interest in mechanical antennas. Existing studies mainly focus on electret based, permanent magnet based, piezoelectric, and magnetoelectric resonant schemes. Electret-based mechanical antennas radiate through the time-varying distribution of bound charges under rotational or vibrational motion [6,7], and recent work has established their radiation models and improved their transmission performance through charge-density enhancement and structural optimization [8,9,10]. Permanent-magnet-based mechanical antennas generate radiation by mechanically modulating magnetic dipole moments [11], with reported advances in harmonic modulation, magnetic-resonance-based transmission, array configurations, and directional communication [12]. Piezoelectric mechanical antennas exploit stress-induced dynamic dipole moments or coupled electromechanical radiation effects [13,14,15], showing promise for compact integration and broader frequency design flexibility. Despite these advances, mechanical antennas still face trade-offs among radiation efficiency, bandwidth, modulation rate, and mechanical complexity. In this context, mechanical magnetoelectric antennas are particularly attractive because they utilize magnetostrictive-piezoelectric coupling to achieve efficient low-frequency radiation while retaining miniaturization potential, thus offering a promising pathway beyond the limitations of existing mechanical antenna technologies.

Mechanical magnetoelectric (ME) antennas provide a promising alternative. Unlike conventional electric antennas, which radiate through the oscillation of free charges, ME antennas generate and detect low-frequency electromagnetic signals through mechanical–electrical–magnetic coupling in functional materials. In the converse magnetoelectric effect (CME), an alternating electric field drives the piezoelectric phase into vibration; the resulting strain is transferred to the magnetostrictive phase, modulating its magnetization and producing a time-varying magnetic dipole moment for radiation [16]. Because the operating frequency is mainly determined by mechanical resonance rather than electromagnetic wavelength, and because acoustic velocities in solids are far lower than the speed of light, ME antennas can operate at low frequencies with dimensions much smaller than those of conventional electric antennas. In the direct magnetoelectric effect (DME), an external alternating magnetic field induces strain in the magnetostrictive phase, which is transferred to the piezoelectric phase and converted into charge or voltage output [17]. Owing to the reciprocity between the direct and converse ME coefficients, the same device can in principle support both reception and transmission, offering a foundation for compact, low-power reciprocal ME antenna systems [18,19,20].

The core of a mechanical ME antenna is a laminated ME composite, whose DME performance depends largely on elastic coupling among the magnetostrictive layer, the piezoelectric layer, and their interface. Ryu et al. reported a PZT/Terfenol-D laminated composite with a magnetoelectric voltage coefficient of 4.7 V/(cm∙Oe), significantly exceeding those of previously reported single-phase and particulate composites [21]. Since then, various laminated systems have been developed, including ferrite/piezoelectric ceramic, magnetic alloy/piezoelectric ceramic, magnetic alloy/piezoelectric single crystal, and magnetic alloy/piezoelectric fiber composites [22]. Among them, Metglas/PMN-PT is especially attractive because Metglas offers high permeability and low coercivity, while PMN-PT provides a large piezoelectric coefficient, enabling both strong magnetostrictive response and efficient piezoelectric conversion.

Among laminated ME architectures, the multi-push-pull (MPP) structure has drawn particular attention because of its strong ME response and flexible structural design. Wang et al. proposed and optimized an MPP composite in which piezoelectric fibers are interconnected through a flexible printed circuit board, allowing multiple piezoelectric units to generate cooperative electrical outputs under strain excitation [23]. With continued optimization of materials and structures, PMN-PT/Metglas MPP devices have achieved a quasi-static charge coefficient of 2700 pC/Oe and, when combined with a low-noise preamplifier, an equivalent magnetic noise of about 5 pT/Hz^1/2^ at 1 Hz, approaching the performance of optically pumped magnetometers, fluxgate magnetometers, and search coils [24]. Based on the CME principle, MPP structures have also been extended to transmitting antennas for low-frequency electromagnetic radiation, creating new opportunities for long-distance wireless communication, wearable devices, and underwater communication [25]. Nevertheless, their complex piezoelectric fiber arrangement, flexible-circuit interconnection, and electrode design still limit fabrication consistency, structural integration, and scalable manufacturing. In addition, equivalent capacitance, dielectric loss, and electromechanical coupling efficiency directly affect both low-noise reception and efficient transmission. For low-frequency ME communication, an ideal antenna should combine high-sensitivity DME reception with strong CME transmission in a single reciprocal device. This makes it important to develop new structures with simpler configurations, lower loss, higher coupling efficiency, and both strong receiving and transmitting capability [26,27].

Recently, in-plane series (IPS) and multilayer laminated ME composites have offered a promising route for further performance improvement. Compared with conventional MPP structures, IPS laminates simplify electrical interconnection, improve strain-transfer pathways, and facilitate device integration. Fang et al. showed that IPS structures exhibit simple fabrication, low dielectric loss, and significantly reduced equivalent capacitance, while achieving a charge coefficient of 3210 pC/Oe at 1 Hz and an equivalent magnetic noise as low as 0.87 pT/Hz^1/2^ at 30 Hz, indicating better magnetic-field detection sensitivity than MPP structures [28]. The reduced capacitance also eases front-end noise matching, while the multilayer laminated configuration is favorable for efficient magnetic–mechanical–electrical energy conversion. However, current studies of IPS structures have focused mainly on magnetic-field detection, and their CME transmission behavior, magnetic-dipole radiation characteristics, and reciprocal transceiver applications remain insufficiently explored [29,30,31].

Against this background, this work investigates the direct and converse magnetoelectric coupling of an IPS Metglas/PMN-PT ME antenna, with particular emphasis on its operating mechanism and advantages as a reciprocal mechanical ME antenna for both reception and transmission. Although IPS-type ME composites with similar fabrication concepts have been reported previously, such as the work by Fang et al. [28], those studies mainly focused on magnetic-field reception based on the direct ME effect. In addition, the number and configuration of the PMN-PT single-crystal fibers used in previous IPS devices differ from those in the present design, leading to differences in device architecture and coupling behavior. More importantly, previous studies primarily provided characterization of the direct ME coupling coefficient and magnetic-field detection capability at relatively high frequencies, while the converse ME response, transmit/receive reciprocity, low-frequency radiation behavior, and system-level transmission capability of IPS-based ME antennas remained insufficiently investigated.

Furthermore, the reciprocal effect discussed in this work refers to the reciprocal transduction between the transmitting and receiving modes of the mechanical magnetoelectric antenna, rather than to a universal reciprocal propagation or transport property of magnetoelectric media. In our antenna, electric excitation induces piezoelectric strain, which is transferred to the magnetostrictive phase to dynamically modulate magnetization and generate electromagnetic radiation, while an incident magnetic field can conversely excite the magnetostrictive phase, produce strain, and generate voltage through the piezoelectric phase. This bidirectional magneto-mechano-electric energy conversion is fundamentally different from non-reciprocal transport phenomena reported in some magnetoelectric or magnetically coupled systems. For example, Chotorlishvili et al. [32]. demonstrated an Fe/BaTiO_3_ multiferroic thermal diode, where interfacial magnetoelectric coupling modifies thermal-excitation transmission and produces direction-dependent heat transport. Similarly, Wang et al. [33] showed that Dzyaloshinskii-Moriya interaction can modify thermally induced magnonic spin currents, generate thermomagnonic torques, and control the velocity and direction of domain-wall motion, enabling DMI-based spin-caloritronic rectification. These studies exploit magnetoelectric or magnetic coupling to achieve non-reciprocal heat or magnonic transport under symmetry-breaking conditions. In contrast, the present antenna does not rely on thermal excitation transport, magnonic spin-current rectification, or domain-wall motion, but uses strain-mediated coupling between magnetostrictive and piezoelectric phases, with mechanical resonance enhancing coherent energy conversion among electric, mechanical, and magnetic domains. Therefore, magnetoelectric coupling should be regarded as a coupling mechanism, while reciprocity or non-reciprocity is determined by the complete dynamical system, including symmetry, energy carriers, boundary conditions, external bias, and operating regime. Thus, previous non-reciprocal transport studies and the present reciprocal mechanical magnetoelectric antenna represent different physical manifestations of magnetoelectric coupling in distinct device contexts.

To fill these gaps, this work provides a systematic investigation of IPS-based ME antennas from device-level coupling mechanisms to system-level wireless transmission performance. First, finite-element simulations are established and validated to analyze both DME and CME responses of the IPS laminate under quasi-static and frequency-domain conditions, including magnetoelectric coupling coefficients, resonant frequencies, and vibration modes. Second, IPS-based low-noise receiving and transmitting antennas are fabricated and experimentally characterized to evaluate their magnetic-field detection capability, converse-ME-driven low-frequency radiation performance, and parallel-array magnetic-field enhancement. Finally, a reciprocal ME wireless communication system is constructed to verify that the IPS ME antenna can function not only as a magnetic-field sensor using the direct ME effect, but also as a transmitting antenna driven by the converse ME effect. Its performance is further directly compared with that of conventional MPP structures in terms of transmission distance, radiated magnetic-field intensity, and system-level integration [34,35,36,37].

## 2. Sample Fabrication and Measurement System

Based on the above research framework, sample fabrication and experimental verification were further carried out. The fabrication process and measurement methods of the IPS-type magnetoelectric antennas are introduced as follows.

**Figure 1:**
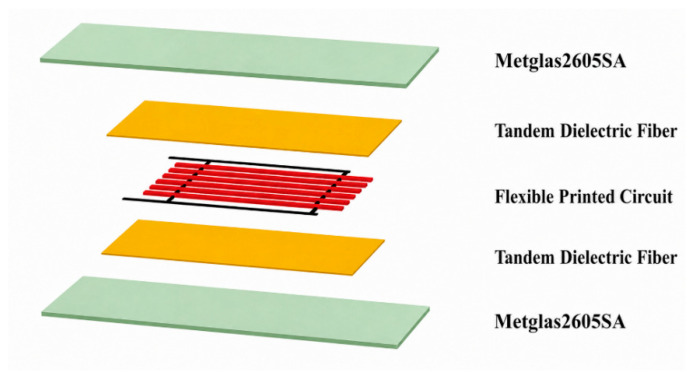
Structural diagram of the IPS-type magnetoelectric antenna.

Figure 1 presents a schematic of the IPS-type magnetoelectric composite structure fabricated in this study. Metglas 2605SA (Qingdao, China) was selected as the magnetostrictive phase and cut into thin foils with dimensions of 140 mm × 20 mm × 0.1 mm. During sample fabrication, every four layers of Metglas foils (Metglas 2605SA) were bonded using West System 105/206 epoxy resin (Beijing, China) and subsequently cured in an oven. Mn-doped PMNT single crystal was employed as the piezoelectric phase, and the crystal was grown by the Bridgman method. The PMNT single crystal was then sliced into wafers, and gold electrodes were deposited on both the upper and lower surfaces. The crystallographic orientation was determined using X-ray diffraction, such that the length, width, and thickness directions of the crystal fibers were oriented along the [001], [110], and [110] directions, respectively. The wafers were further diced into piezoelectric crystal fibers with dimensions of 40 mm × 2 mm × 0.5 mm. After dicing, the samples were poled under an electric field of 10 kV/cm for 5 min.

The poled PMNT crystal fibers (Shanghai, China) were bonded onto a flexible interdigitated electrode (ID-electrode) using prefabricated Stycast 1264 PTA/PTB epoxy resin (Beijing, China) according to the designed arrangement, thereby realizing electrical series connection among the piezoelectric units. Finally, the pre-bonded and cured four-layer Metglas laminates were attached to the upper and lower surfaces of the series-connected piezoelectric core layer using West System 105/206 epoxy resin (Beijing, China) and cured under vacuum conditions, yielding the IPS-type laminated magnetoelectric composite samples.

**Figure 2:**
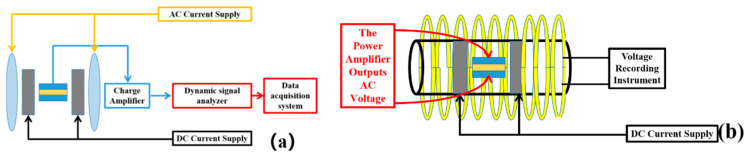
Test flowcharts for (**a**) the direct magnetoelectric effect and (**b**) the converse magnetoelectric effect.

The schematic diagram of the direct magnetoelectric effect measurement system is shown in Figure 2a. During the measurement, a precision current source (Keithley 6221, Cleveland, OH, USA) was first used to drive a Helmholtz coil to apply a small-signal AC magnetic field of $H_{\mathrm{ac}}\;=\;0.1$ Oe along the length direction of the magnetoelectric composite. Meanwhile, a DC current source (Instek PPE3323, Suzhou, China) was used to drive an electromagnet to apply a DC bias magnetic field along the same direction. The optimal operating point was obtained by adjusting the bias magnetic field. The charge signal generated at the two terminals of the magnetoelectric composite was collected using a charge amplifier (Brüel & Kjær 2637, Copenhagen, Denmark) and then fed into a dynamic signal analyzer (Agilent 37670A, Murrieta, CA, USA) for real-time monitoring and data acquisition.

The schematic diagram of the converse magnetoelectric effect measurement system is shown in Figure 2b. During the test, the electromagnet was first driven by a DC current source to provide the optimal DC bias magnetic field for the sample. Subsequently, an 80-turn induction coil was tightly wound around the outer surface of the magnetoelectric composite, and an AC driving voltage $V_{\mathrm{in}}$ was applied across the two terminals of the composite through a power amplifier. Under AC voltage excitation, the piezoelectric phase underwent periodic mechanical deformation, which drove the magnetostrictive phase to vary its magnetization through interfacial strain coupling. As a result, an alternating magnetic field was generated around the sample based on the converse magnetoelectric effect. This alternating magnetic field further induced an AC output voltage $V_{\mathrm{out}}$ in the induction coil, and the real-time signal was used to calculate the converse magnetoelectric response of the sample [38].

(1) $$U=V_{\mathrm{out}}\sin(\omega t)=\frac{-N\Delta \phi }{\Delta t}=-NA_{m}\frac{\Delta B}{\Delta t}$$

(2) $$\alpha_{\mathrm{CME}}=\frac{\partial B}{\partial V}$$

(3) $$V={\;V}_{\mathrm{in}}\cos\left(\omega t\right)$$

(4) $$\alpha_{\mathrm{CME}}=\frac{\partial B}{\partial V}=\frac{\partial B}{\partial t}\frac{\partial t}{\partial V}=\frac{V}{\omega NA_{m}V_{\mathrm{in}}}$$

## 3. Finite Element Simulation and Analysis

After presenting the sample fabrication procedure and measurement system, finite element simulations were performed to further analyze the internal coupling behavior of the devices, thereby theoretically revealing their performance differences and enhancement mechanisms. With the development of multiphysics numerical modeling techniques, COMSOL 5.6 (2021, Sweden) finite-element simulation has become an important tool for analyzing the response behavior and coupling mechanisms of magnetoelectric composites. By establishing an appropriate finite-element model, the strain-transfer process between the magnetostrictive and piezoelectric phases, the magnetic–mechanical–electrical energy conversion mechanism, and the vibration modes of the device at different operating frequencies can be further elucidated. To ensure the systematicity and completeness of the subsequent investigation, COMSOL was employed in this work to numerically simulate the direct and converse magnetoelectric effects of the Metglas/PMNT laminated magnetoelectric composite, predict its magnetoelectric coupling coefficients, and analyze the corresponding operating modes. The material parameters of Metglas used in the simulations are listed in Table 1 [39].

**Figure 3:**
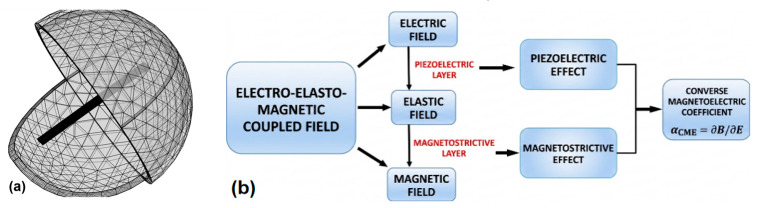
(**a**) Structural meshing for finite element simulation and (**b**) the flowchart of multiphysics coupling.

As shown in Figure 3a, a laminated magnetoelectric composite structure was established in this work based on the finite-element method. The model consists of upper and lower magnetostrictive Metglas layers and a middle piezoelectric PMNT single-crystal layer. The length, width, and thickness of each Metglas layer are 140 mm, 20 mm, and 0.1 mm, respectively, while those of the PMNT single-crystal layer are 40 mm, 20 mm, and 0.5 mm, respectively. To reduce the influence of boundary conditions on the magnetic-field distribution and simulation results, a sufficient air domain was constructed around the magnetoelectric composite with a radius of 2000 mm. The total number of mesh elements was set to 28,000 to balance computational stability and result accuracy [40]. In Figure 3b, the fundamental principles of the direct and converse magnetoelectric effects, magnetostrictive materials undergo corresponding deformation under an applied magnetic field as the magnetic flux density or magnetization state changes. Similar to typical magnetostrictive materials such as Terfenol-D and Galfenol, magnetoelectric composites generally require an optimized DC bias magnetic field during operation, so that the magnetostrictive phase works within an approximately linear response region. Therefore, in the simulation model of this study, the deformation of the magnetostrictive layer can be approximately regarded as being within the linear elastic regime [41].

To simplify the model and improve computational efficiency, the Metglas magnetostrictive layer was assumed to be an isotropic material. Here, C denotes the stiffness matrix determined jointly by Young’s modulus and Poisson’s ratio; T is the stress generated in the magnetostrictive layer under multiphysics coupling; $T_{0}$ represents the initial stress; m is the magnetostrictive strain tensor; ∇ is the gradient operator; and the superscript T denotes matrix transposition. In this formulation, $\lambda_{s}$ is the saturation magnetostriction coefficient, $M_{s}$ is the saturation magnetization, dev denotes the deviatoric tensor operator, and $M_{i}M_{j}$ represents the tensor product between magnetization components. The governing equations for the magnetostrictive effect in the finite-element model are given as follows:

(5) $$S_{3,m}\;=\;\frac{3}{2}\lambda_{s}\frac{M_{i}M_{j}}{M^{2}}\;=\;\frac{3}{2}\lambda_{s}{\left(\frac{M}{M_{s}}\right)}^{2}$$

(6) $$T_{3,m}=C_{H}[S_{0}-S_{3,m}(M)]$$

(7) $$=M_{s}L(\frac{3\chi_{0}H_{\mathrm{eff}}}{M_{s}})$$

(8) $$=\coth(\frac{3\chi_{0}H_{\mathrm{eff}}}{\mathrm{Ms}})-\frac{M_{s}}{3\chi_{0}H_{\mathrm{eff}}}$$

The dynamic equilibrium equation and the frequency-domain governing equations of the laminated magnetoelectric composite are given as follows:

(9) $$\frac{\partial^{2}u}{\partial t^{2}}\;=\;\nabla T\;+\;F_{v}$$

(10) $$-\rho \omega^{2}u=\nabla T+F_{v}e^{i\varphi }$$

where i denotes different material phases, with m and p representing the magnetostrictive and piezoelectric phases, respectively; ρ is the material density, u is the displacement vector, T is the stress tensor, and $F_{v}$ represents the sum of body forces.

In general, an obvious nonlinear relationship between strain and electric field appears only under strong electric-field excitation. Since the driving voltage applied in this work is relatively low, the piezoelectric phase still operates within the linear response regime; thus, the relationship between strain and electric field can be approximately regarded as linear. Here, S denotes strain, $S_{0}$ denotes initial strain, $s^{E}$ is the compliance coefficient matrix under a constant electric field, d is the piezoelectric coefficient matrix, E and D represent the electric-field intensity and electric displacement vector, respectively, and ε is the permittivity matrix. The governing equations for the piezoelectric effect in the finite-element model are as follows:

(11) $$S-S_{0}\;={\;s}^{E}(T-T_{0})\;+\;d^{T}E$$

(12) $$D-D_{r}=d(T-T_{0})+\xi E$$

(13) $$\nabla \cdot D=\rho_{v}$$

(14) E=−∇V

In this model, the bottom surface of the piezoelectric layer was grounded, and the external circuit was set to an open-circuit condition. The initial conditions are as follows:

(15) $$D_{z}\;=\;0$$

(16) $$u_{x=0}=0$$

In magnetoelectric composites, the direct magnetoelectric coupling coefficient $\alpha_{Q}$ or $\alpha_{ME}$ is commonly used to characterize the coupling response between the magnetostrictive and piezoelectric phases under an applied magnetic field, namely, the process in which the magnetostrictive phase generates strain under magnetic-field excitation and induces an electric field or voltage output in the piezoelectric phase through interfacial stress transfer. The converse magnetoelectric coefficient characterizes the magnetic response induced by an applied electric field. In strain-mediated magnetoelectric composites, an external electric field first drives deformation in the piezoelectric phase, and the resulting strain is transferred to the magnetostrictive phase through the interface, thereby inducing a change in magnetization or equivalent magnetic field. Therefore, the converse magnetoelectric coefficient is commonly defined as dH/dE.

(17) $$\alpha_{Q}=\frac{\partial Q}{\partial H}$$

(18) $$\alpha_{\mathrm{CME}}=\frac{\partial H}{\partial E}$$

Based on the above finite-element governing equations and model construction, the direct magnetoelectric effect (DME) of the composite was first analyzed under steady-state conditions. DC bias magnetic field $H_{\mathrm{dc}}$, was applied along the Y-axis, and a parametric sweep was performed in the COMSOL stationary solver with $H_{\mathrm{dc}}$ varying from 0 to 30 Oe. The magnetostrictive strain, piezoelectric terminal voltage, and magnetic flux density distribution were then systematically evaluated as functions of the bias magnetic field.

**Figure 4:**
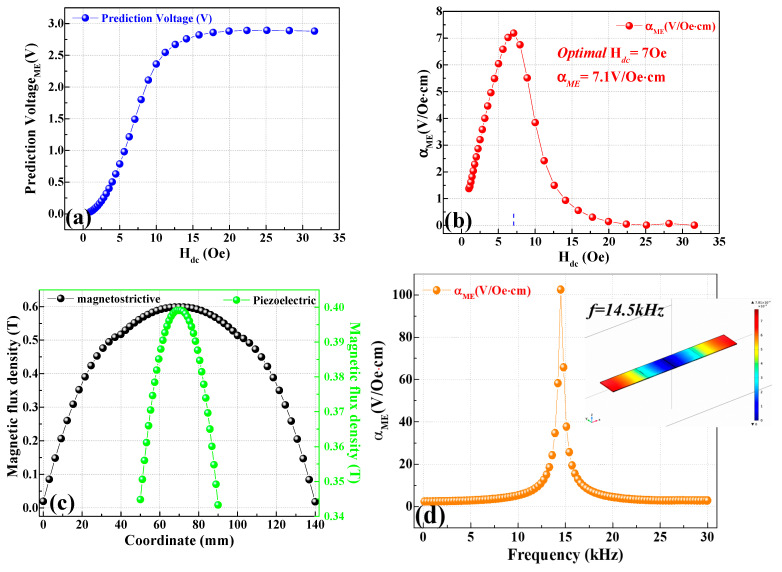
(**a**) Induced voltage with H_dc_; (**b**) α_DME_ with H_dc_ curve; (**c**) magnetic flux density along the length of the composite; (**d**) α_DME_ with frequency.

Figure 4 summarizes the simulated DME response of the IPS-type Metglas/PMNT laminated magnetoelectric composite, including the quasi-static bias-field response, magnetic flux density distribution, and frequency-domain resonance characteristics. As shown in Figure 4a, with increasing $H_{\mathrm{dc}}$, the longitudinal strain of the magnetostrictive layer increases continuously and approaches saturation near $H_{\mathrm{dc}}\;=\;20$ Oe, with a saturation strain of approximately $\lambda_{s}\;=\;27$ ppm. Meanwhile, the terminal voltage generated by the piezoelectric phase also increases and eventually reaches a maximum of $V_{\max}\;=\;2.7$ V. This behavior arises from magnetic-domain rotation and enhanced magnetization in the Metglas layer under the applied DC field, which induce longitudinal magnetostrictive deformation. The resulting strain is transferred to the piezoelectric layer through interfacial stress, generating a voltage output via the piezoelectric effect. As the magnetostrictive phase approaches magnetic saturation, the incremental strain decreases, and the piezoelectric output correspondingly tends toward saturation.

According to the relationship between the terminal voltage of the piezoelectric layer and the applied magnetic field, the quasi-static DME coupling coefficient $\alpha_{\mathrm{DME}}$ as a function of $H_{\mathrm{dc}}$ was calculated, as shown in Figure 4b. The result exhibits a typical increase-then-decrease trend. At $H_{\mathrm{dc}}\;=\;7$ Oe, the magnetoelectric coupling coefficient reaches its maximum, with $\alpha_{\mathrm{ME}}\;=\;7.1$ V/(Oe·cm). This indicates that, in the low-bias-field region, the magnetostrictive layer is highly sensitive to changes in the applied magnetic field, corresponding to a large piezomagnetic coefficient and strong magnetic-to-mechanical conversion. As the bias field further increases toward saturation, the differential strain response weakens, leading to a reduction in the magnetoelectric coupling coefficient. Therefore, an optimal bias field exists at which the strain sensitivity of the magnetostrictive phase, the interfacial stress-transfer efficiency, and the electrical output capability of the piezoelectric phase are best matched. This result provides an important basis for bias-field optimization in the receiving unit of a mechanical magnetoelectric antenna.

Figure 4c further presents the magnetic flux density distribution along the length direction of the magnetostrictive and piezoelectric layers under the optimal bias field of $H_{\mathrm{dc}}\;=\;7$ Oe. The magnetic flux density is approximately symmetrically distributed and reaches its maximum in the central region of the structure. The flux density in the magnetostrictive layer is significantly higher than that in the piezoelectric layer, mainly because of the different magnetic responses of the two phases. As a high-permeability soft magnetic material, the flux density in Metglas is governed by its magnetization curve $\left(M-H\right)$, whereas the PMNT piezoelectric single crystal is a nonmagnetic or weakly magnetic medium whose magnetic flux density is primarily determined by $B\;={\;\mu }_{0}\mu_{r}H$ [42]. In addition, the higher and more concentrated magnetic flux density in the central region of the magnetostrictive layer indicates stronger magnetic-field concentration and magnetostrictive response there. Since the PMNT layer is located near the center of the composite, this configuration facilitates strain-energy transfer from the magnetostrictive phase to the piezoelectric phase, thereby improving the overall magnetoelectric conversion efficiency.

On the basis of the quasi-static analysis, the frequency-domain DME response of the IPS-type magnetoelectric composite was further investigated. In this simulation, the DC bias field was fixed at the optimal value of $H_{\mathrm{dc}}\;=\;7$ Oe, and an AC magnetic field with an amplitude of $H_{\mathrm{ac}}\;=\;1$ Oe was applied. As shown in Figure 4d, $\alpha_{\mathrm{ME}}$ exhibits a pronounced nonlinear frequency dependence. In the low-frequency non-resonant region, the magnetoelectric coupling coefficient remains relatively low. When the frequency approaches 14.5 kHz, however, $\alpha_{\mathrm{ME}}$ increases sharply and exhibits a distinct resonance peak, reaching approximately 110 V/(Oe·cm), which is much higher than that under quasi-static conditions. Combined with the vibration-mode distribution shown in the inset, this peak can be attributed to the longitudinal extensional vibration mode of the composite structure. Under this mode, the interfacial strain coupling between the magnetostrictive and piezoelectric layers is greatly enhanced, and mechanical energy is efficiently accumulated, leading to significant amplification of the magnetic–mechanical–electrical conversion process.

In summary, the designed Metglas/PMNT laminated magnetoelectric composite exhibits strong direct magnetoelectric coupling under the optimal DC bias field of $H_{\mathrm{dc}}\;=\;7$ Oe and a pronounced resonance enhancement near 14.5 kHz. For the receiving unit of a mechanical magnetoelectric antenna, these results indicate that efficient operation can be achieved by properly optimizing the bias field and operating frequency, thereby enabling highly sensitive reception of weak low-frequency magnetic-field signals. In particular, operation near resonance allows the composite structure to overcome the size-limited radiation and reception efficiency of conventional electrically small antennas in the low-frequency range, providing theoretical support for the development of miniaturized, low-power, and highly sensitive mechanical magnetoelectric antennas.

**Figure 5:**
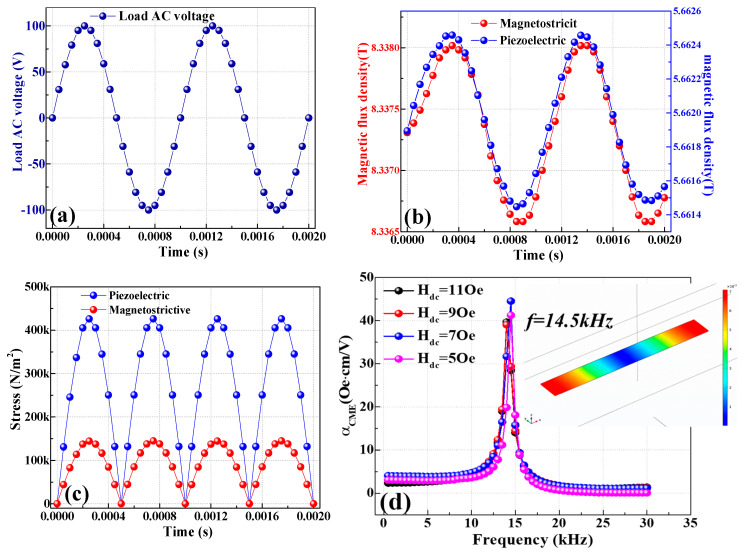
(**a**) Piezoelectric phase voltage as a function of time; (**b**) magnetic flux density versus time through magnetostrictive and piezoelectric phases; (**c**) the curve of Stress through magnetostrictive and piezoelectric phases with time; (**d**) converse ME coefficient versus frequency under different DC bias magnetic fields.

Figure 5 presents the simulated converse magnetoelectric effect (CME) of the IPS-type laminated magnetoelectric composite based on the same finite-element model, focusing on the electric-mechanical-magnetic coupling process under AC voltage excitation and its frequency-domain resonance behavior. These results correspond to the transmitting mode of the mechanical magnetoelectric antenna, in which the input electrical signal is converted into mechanical vibration through the converse piezoelectric effect and subsequently into an alternating magnetic response through strain-mediated coupling in the magnetostrictive phase.

As shown in Figure 5a, a voltage was applied across the piezoelectric phase, inducing periodic extensional deformation. The generated strain and stress were transferred to the magnetostrictive phase through the interface, establishing the fundamental electric-to-mechanical conversion pathway of the CME. Figure 5b shows that the magnetic flux density in both phases varies periodically under AC excitation. In the magnetostrictive phase, this variation arises from stress-induced magnetization modulation, indicating that the mechanical vibration generated by the piezoelectric layer can be effectively converted into magnetic energy output. Figure 5c further shows that both phases exhibit periodic stress oscillations, although the stress amplitude is significantly higher in the piezoelectric phase. This confirms that the piezoelectric layer acts as the active driving component, whereas the magnetostrictive layer serves as the passive magnetic-response layer, and also highlights the importance of interfacial strain-transfer efficiency for CME performance.

Figure 5d further illustrates the dependence of the converse magnetoelectric coupling coefficient $\alpha_{\mathrm{CME}}$, on frequency and DC bias magnetic field $H_{\mathrm{dc}}$. Under different bias fields, $\alpha_{\mathrm{CME}}$ exhibits a pronounced resonance peak near 14.5 kHz, which corresponds to the longitudinal extensional vibration mode of the composite. At resonance, the internal vibration amplitude and interfacial strain coupling are significantly enhanced, leading to efficient electric-mechanical-magnetic energy conversion. The DC bias field also strongly affects the CME response, and the maximum value, $\alpha_{\mathrm{CME}}\;\approx \;45$ Oe·cm/V, is obtained at $H_{\mathrm{dc}}\;=\;7$ Oe. This optimal bias is consistent with that of the direct magnetoelectric effect (DME), indicating that the same bias condition can simultaneously optimize both receiving and transmitting performance. Physically, at $H_{\mathrm{dc}}\;=\;7$ Oe, the magnetostrictive phase operates in a high-piezomagnetic-response region, making its magnetization more sensitive to stress perturbation and thereby enhancing magnetic-flux-density modulation. When the bias field deviates from this value, the piezomagnetic coefficient decreases, resulting in weaker stress-to-magnetization conversion and a reduced $\alpha_{\mathrm{CME}}$.

**Figure 6:**
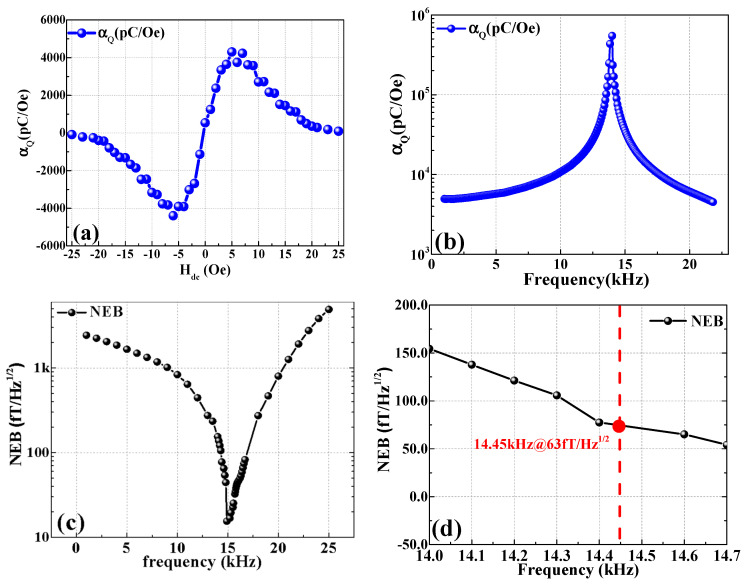
(**a**) The relationship between charge coefficient and DC bias magnetic field H_dc_. (**b**) The curve of measured charge coefficient with frequency. (**c**) The curve of NEB with frequency. (**d**) Detail-magnified view.

As shown in Figure 6a, α_Q_ exhibits a pronounced nonlinear dependence on H_dc_. In the positive bias-field region, α_Q_ increases rapidly with increasing, reaches a maximum of about 4300 pC/Oe near 7 Oe, and then gradually decreases. This behavior mainly arises from the bias dependence of the piezomagnetic coefficient of the Metglas layer. At low bias fields, domain rotation and magnetization are highly sensitive to the applied AC magnetic field, resulting in enhanced magnetostrictive strain variation. As the bias field approaches magnetic saturation, however, the differential strain response weakens, leading to reduced magnetoelectric conversion efficiency. In addition, the static coupling coefficient shows an approximately antisymmetric behavior under positive and negative bias fields, indicating that the output polarity is closely related to the magnetization direction of the magnetostrictive phase. These results agree well with the finite-element prediction and confirm that 7 Oe is the optimal operating bias.

Figure 6b shows the frequency response of α_Q_ under the optimal bias field. Away from resonance, the magnetoelectric response remains relatively low, whereas a sharp resonance peak appears at 14.1–14.5 kHz, with a maximum value of approximately 340 kpC/Oe. Compared with the non-resonant state, the charge output is enhanced by several orders of magnitude, indicating that the structure enters an electromechanical resonance state in this frequency range. At resonance, the weak-field-induced magnetostrictive strain is strongly amplified by the mechanical quality factor and efficiently transferred to the piezoelectric phase, producing a large charge output. Consistent with the simulation results, this peak corresponds to the first-order longitudinal extensional mode, demonstrating good agreement between experiment and finite-element analysis in both resonant frequency and vibration mode.

To further evaluate the weak magnetic-field detection capability, Figure 6c,d present the NEB (equivalent magnetic-field noise) spectra measured in a magnetically shielded environment. As a key indicator of sensor sensitivity, a lower NEB corresponds to a lower detectable magnetic-field limit. Figure 6c shows that the NEB decreases significantly with frequency and reaches its minimum near resonance, consistent with the ME effect.

The *α_Q_* in Figure 6d further shows that the minimum equivalent magnetic noise density reaches 63 fT/Hz^1/2^ near 14.45 kHz. This result demonstrates that the IPS magnetoelectric receiving antenna can achieve highly sensitive room-temperature detection of extremely weak low-frequency magnetic signals. Moreover, the concentration of minimum NEB values near resonance indicates not only high sensitivity but also strong frequency selectivity, enabling enhancement of target-frequency signals while suppressing off-resonance noise and interference, and thus providing an inherent narrowband filtering capability.

**Figure 7:**
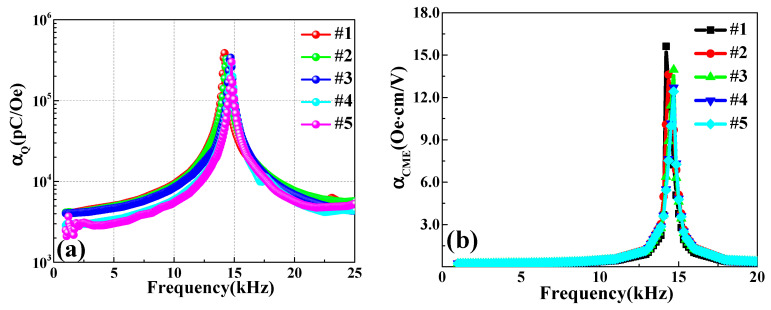
The curve of Five IPS composite: (**a**) α_DME_ with frequency; (**b**) α_CME_ with frequency.

Figure 7 compares the frequency-domain DME and CME responses of five IPS-type laminated magnetoelectric composite cores with identical dimensions (#1–#5) to evaluate device consistency, reproducibility, and suitability for array integration in both receiving and transmitting modes. As shown in Figure 7a, under the optimal DC bias field, all five samples exhibit typical resonance-enhanced DME responses. Away from resonance, the magnetoelectric charge coefficient $\alpha_{Q}$ remains relatively low, whereas sharp peaks appear at 14.1–14.5 kHz, consistent with the finite-element predictions and single-device measurements. This confirms that the resonance originates from the first-order longitudinal extensional mode of the IPS structure, in which AC magnetic excitation induces magnetostrictive strain that is transferred to the piezoelectric layer, resulting in a strongly enhanced charge output. Sample #1 shows the highest peak $\alpha_{Q}$ of about 3.4 × 10^6^ pC/Oe, while the other samples fall within 3.0 × 10^6^ to 3.4 × 10^6^ pC/Oe.

Figure 7b shows the corresponding CME responses of the same five samples in transmitting mode. All samples exhibit pronounced resonance peaks at 14.1–14.5 kHz, with resonant frequencies highly consistent with those observed in the DME measurements. This indicates that the periodic extensional vibration generated in the piezoelectric layer under AC voltage excitation can be effectively transferred to the magnetostrictive layer, where it induces alternating magnetization through the inverse magnetostrictive effect, thereby enabling efficient electric-mechanical-magnetic conversion. The peak $\alpha_{\mathrm{CME}}$ values are concentrated in the range of 12.0–15.8 Oe·cm/V, with sample #1 again exhibiting the highest value of about 15.8 Oe·cm/V. These results demonstrate that the IPS composite structure offers both high receiving sensitivity and efficient low-frequency magnetic-field transmission, while also showing good device-to-device consistency for array applications.

**Figure 8:**
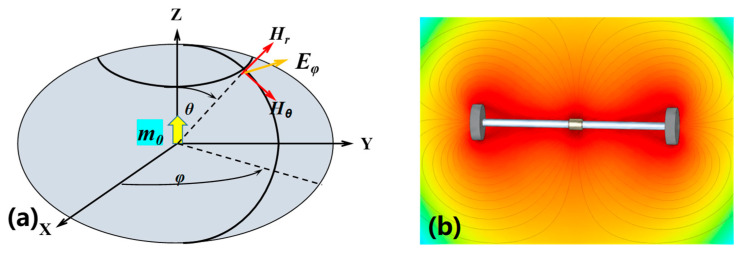
(**a**) Theoretical spatial electromagnetic field distribution and (**b**) simulation results.

In addition, the ME antenna can be equivalently modeled as a magnetic dipole antenna, and the equivalent dipole radiation model can be used to analyze its radiation characteristics. As shown in Figure 8a, a spherical coordinate system is established with the center of the magnetic dipole as the origin. The coordinates of point P are denoted as (r, θ, φ), where r represents the distance from the magnetic dipole, and θ and φ denote the angular coordinates in the spherical coordinate system. The results indicate that as the distance increases, the radiation distribution of the magnetic dipole gradually expands outward, while the radiation intensity decays more significantly with increasing distance. The radiation pattern resembles a “∞” shape and exhibits stronger radiation capability along specific directions. In Figure 8b, the near-field radiation pattern of the magneto-electric antenna was also simulated, which is consistent with the characteristics of the magnetic dipole.

The magnetic and electric field components of a magnetic dipole can be expressed as follows, where $p_{m}$ denotes the magnetic dipole moment [43,44,45,46].

(19) $$\left\{\begin{array}{c} H_{r}=\frac{k^{3}p_{m}}{2\pi \mu }\left(\frac{j}{{\left(\mathrm{kr}\right)}^{2}}+\frac{1}{{\left(\mathrm{kr}\right)}^{3}}\right)e^{-\mathrm{jkr}}\cos\theta \\ H_{\theta }=-\frac{k^{3}p_{m}}{4\pi \mu }\left(\frac{1}{\mathrm{kr}}-\frac{j}{{\left(\mathrm{kr}\right)}^{2}}-\frac{1}{{\left(\mathrm{kr}\right)}^{3}}\right)e^{-\mathrm{jkr}}\sin\theta \\ E_{\varphi }=\frac{\omega k^{2}p_{m}}{4\pi }\left(\frac{1}{\mathrm{kr}}-\frac{j}{{\left(\mathrm{kr}\right)}^{2}}\right)e^{-\mathrm{jkr}}\sin\theta \end{array}\right.$$

Because the ME antenna operates on the basis of the magnetoelectric coupling effect, it primarily senses the magnetic-field component of the electromagnetic wave. In the near-field region (r≪λ), where kr≪1 and $e^{-\mathrm{jkr}}\;\approx \;1$, the magnetic-field component in Equations (20) and (21) can be written as:

(20) $$H_{r}\;=\;\frac{p_{m}\cos\theta }{2\pi \mu r^{3}}$$

(21) $$H_{\theta }=\frac{p_{m}\sin\theta }{4\pi \mu r^{3}}$$

According to the above expressions, the magnetic-field component of the near-field radiation generated by an ME antenna decays with the cube of the distance.

**Figure 9:**
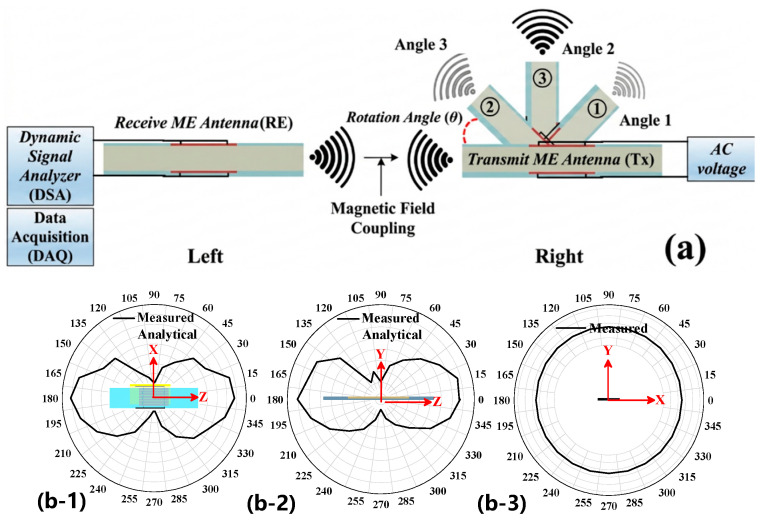
(**a**) schematic diagram of angle relationship test between antenna emitting end and ME sensor; (**b-1**–**b-3**) the spatial radiation angle of the ME antenna.

Figure 9 presents the angular coupling test results between the transmitting unit of the mechanical magnetoelectric antenna and the magnetoelectric receiving antenna, aiming to evaluate the effect of relative orientation between the transmitter and receiver on the transmission efficiency of low-frequency magnetic-field signals. As shown in Figure 9a, one IPS-type magnetoelectric antenna was used as the transmitter and another IPS magnetoelectric antenna was used as the receiver, with the transmission distance fixed at 3 m. The transmitter was driven by an AC voltage signal with an amplitude of 50 V and a frequency of 14.45 kHz, corresponding to the structural resonant frequency obtained from the aforementioned DME/CME measurements. To reduce the influence of electric-dipole near-field coupling and ambient electric-field noise on the test results, the outer surface of the IPS magnetoelectric receiving antenna enclosure was wrapped with copper foil for electric-field shielding. This treatment effectively suppresses spurious voltage output caused by electric-field crosstalk, ensuring that the received signal mainly originates from the coupling of the alternating magnetic field generated by the transmitting antenna. Therefore, this experiment can more accurately reflect the actual magnetic coupling characteristics of the mechanical magnetoelectric antenna in a low-frequency magnetic communication link.

In the angular test, the included angle between the transmitter and receiver was defined as 0° when they were placed in parallel. The relative angle was then varied in steps of 15° by rotating either the transmitter or the receiver, and the output voltage amplitude of the receiver was recorded at each angle. The polar plot in Figure 9b shows the variation in the received voltage with rotation angle, together with a comparison between the experimental results and theoretical predictions. It can be seen that the received signal amplitude exhibits pronounced directional characteristics with angle. When the transmitter and receiver are in the parallel or antiparallel configurations, i.e., at 0° and 180°, the receiver output voltage reaches its maximum value of approximately 25 μV. In contrast, when the included angle is 90° or 270°, the output voltage decreases to its minimum value of approximately 8.3 μV. These results indicate that the alternating magnetic field generated by the IPS magnetoelectric transmitting antenna exhibits typical anisotropic spatial distribution characteristics, and that the received signal intensity is closely related to the relative angle between the magnetic-sensitive axes of the transmitter and receiver.

**Figure 10:**
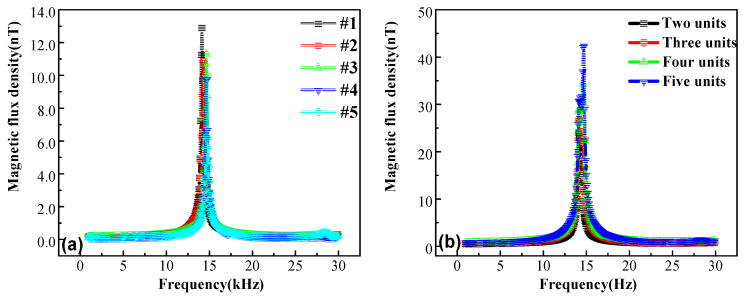
(**a**) Unit emission performance of 5 IPS-type magneto-electric antennas. (**b**) Performance when a single layer is superimposed to form an array.

Figure 10a presents the individual radiation responses of samples #1~#5 over the frequency range of 0.5~30 kHz. It can be observed that all five antennas exhibit distinct resonant characteristics, and their first-order resonant frequencies are highly consistent, all concentrated near 14.5 kHz. This indicates that the fabricated devices possess good consistency and repeatability, which is also an important prerequisite for achieving effective superposition of radiation signals in multi-element operation. Meanwhile, the peak magnetic flux density values of the individual antennas at resonance exhibit some variation, ranging from approximately 9.6 to 13.1 nT. This difference may be attributed to factors such as interfacial bonding quality between the piezoelectric and magnetostrictive layers in the multiferroic composite structure, intrinsic material-property variations, and small errors introduced during fabrication and assembly. In Figure 10b, the five magnetic-electric antenna samples are stacked one after another. The radiation performance of the last five electrically connected magnetoelectric antennas can reach 43 nT.

**Figure 11:**
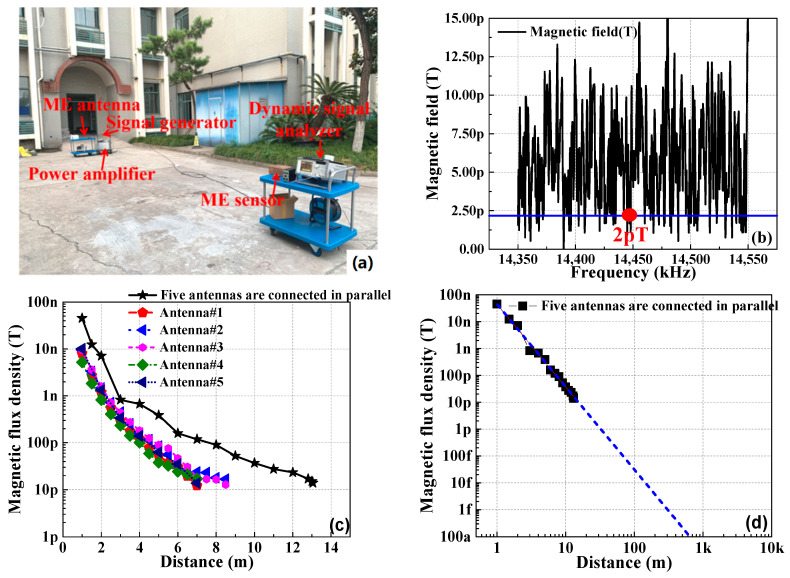
(**a**) Measured image of the ME antenna in the real environment; (**b**) environmental background noise in space; (**c**) relationship between test distance and antenna; (**d**) prediction of the long-distance performance of the ME antenna.

On this basis, to further evaluate the long-distance transmission capability of the IPS ME antenna, the position of the transmitter was fixed, while the separation distance between the receiving sensor and the transmitter was varied, and the signal propagation characteristics under different transmitting configurations were tested. In the experiment, under an AC voltage of 70 V and a frequency of 14.45 kHz, magnetic signals were transmitted using either a single antenna (sample #1, #2, #3, #4, or #5) or five samples connected in parallel, and the maximum distance at which the sensor could still receive an effective signal was measured. As shown in Figure 11a, the relationship between the magnetic signal strength received by the sensor and the transmission distance is presented. The experimental results show that, when a single IPS magnetoelectric antenna is used for transmission, the maximum test distance can reach 8.5 m. At the same receiving position, for example at 1 m, the received magnetic flux density intensity for the five antennas connected in parallel is approximately five times that of a single magnetoelectric antenna. This indicates that parallel operation of multiple antennas can significantly increase the total magnetic dipole moment, thereby enhancing both the magnetic-field radiation intensity at the transmitter and the signal amplitude at the receiver. These results demonstrate that a parallel architecture is an effective strategy for improving the long-distance transmission capability of magnetoelectric antennas.

Figure 11b shows the measured results of the disturbance magnetic field strength in the external environment. It can be seen that the average noise at 14.45 kHz is approximately 2 pT, and this background noise limits, to some extent, the effective receiving capability of the sensor for the transmitted antenna signal. In Figure 11c, the distances that five antenna units can propagate in the real air are given. The radiation performance of the array formed by the parallel connection of these five antenna units can reach 43 nT @ 1 m. The spatial radiation intensity decays approximately in a cubic relationship with the distance, and it can achieve a propagation distance of 13.5 m in the air. In Figure 11d, we have predicted the long-distance radiation capability of the magnetoelectric antenna array. At a frequency of 1 fT, the propagation distance can reach 310 m.

To more intuitively demonstrate the overall performance of the proposed device, Table 2 compares the key metrics of the parallel IPS mechanical magnetoelectric antenna proposed in this work with those of MPP-structured devices reported in the literature. It can be seen that, in terms of transmitting performance, the parallel IPS antenna achieves a magnetic flux density of 37 nT at 1 m, which is significantly higher than the 2 nT of the MPP structure. In terms of propagation capability, its propagation distance reaches 310 m at 1 fT, clearly outperforming the 200 m of the MPP structure. In terms of receiving performance, its equivalent magnetic noise at 14.45 kHz is 63 fT/Hz^1/2^, which is complemented the deficiency in the resonant performance testing of the ME antenna.

## 4. Conclusions

This work systematically investigates the IPS-type Metglas/PMN-PT laminated magnetoelectric composite through theoretical modeling, device fabrication, and experimental characterization, and clarifies its operating mechanism and performance advantages as a reciprocal mechanical magnetoelectric antenna for both transmission and reception. Finite-element results show that, under the optimal DC bias magnetic field, both the DME and CME exhibit strong coupling responses with pronounced resonance enhancement near 14.5 kHz, governed by the same longitudinal extensional mode. These results confirm the excellent transmit–receive reciprocity of the device and provide guidance for structural optimization and operating-point selection. In addition, the strong consistency of the broadband DME and CME responses among five independently fabricated samples demonstrates good fabrication stability, reproducibility, and potential for array integration.

Angular coupling measurements further show that the relative orientation between the transmitting and receiving units strongly affects signal-transfer efficiency, with the parallel configuration producing the strongest magnetic coupling, consistent with the directional characteristics of low-frequency magnetic-dipole radiation and reception. Moreover, parallel excitation of multiple IPS antennas enables an approximately linear increase in equivalent magnetic moment, thereby enhancing the radiated magnetic flux density and extending the transmission distance of low-frequency magnetic signals. Overall, the proposed IPS laminated magnetoelectric composite simultaneously improves receiving sensitivity and transmitting capability while offering miniaturization, low power consumption, reciprocity, and array scalability. These findings provide both a promising device architecture and theoretical support for applications in VLF/ULF wireless communication, underwater communication, and weak magnetic-field detection. Future work should focus on packaging, cooperative array excitation, impedance matching, and communication-performance evaluation in complex environments to advance practical deployment.

## Figures and Tables

**Table 1:** Performance parameters of magnetostrictive Metglas.

Symbol	Description	Unit	Value
δ_M_	Conductivity	S/m	1.3 × 10^6^
ε_r_	Dielectric constant	1	1
E_s_	Young modulus	GPa	110
ν	Poisson ratio	1	0.37
ρ_m_	Density of Metglas	kg/m^3^	7180
Ms	Saturation magnetization	A/m	1.5 × 10^6^
λ_s_	Saturation magnetostriction	ppm	30
μ_0_	Permeability of vacuum	H/m	4π × 10^−7^

**Table 2:** Performance comparison between MPP and IPS.

Model	MPM [37]	MPM [47]	MPP [34]	Parallel Connection IPS
Magnetic flux density	2 nT @ 1 m	10 nT @ 1 m	2 nT @1 m	43 nT @ 1 m
Propagation distance at 1 fT	100 m	215 m	200 m	310 m
equivalent magnetic noise floor	none	none	5.1 pT/Hz^1/2^ @ 1 Hz	63 fT/Hz^1/2^ @ 14.45 kHz

## Data Availability

The original contributions presented in this study are included in the article. Further inquiries can be directed to the corresponding authors.
